# Severe Obstetric Brachial Plexus Palsies Can Be Identified at One Month of Age

**DOI:** 10.1371/journal.pone.0026193

**Published:** 2011-10-17

**Authors:** Martijn J. A. Malessy, Willem Pondaag, Lynda J.-S. Yang, Sonja M. Hofstede-Buitenhuis, Saskia le Cessie, J. Gert van Dijk

**Affiliations:** 1 Department of Neurosurgery, Leiden University Medical Center, Leiden, the Netherlands; 2 Department of Neurosurgery, University of Michigan Hospitals, Ann Arbor, Michigan, United States of America; 3 Department of Neurosurgery Physical Therapy, Leiden University Medical Center, Leiden, the Netherlands; 4 Department of Neurosurgery Medical Statistics, Leiden University Medical Center, Leiden, the Netherlands; 5 Department of Neurosurgery Neurology and Clinical Neurophysiology, Leiden University Medical Center, Leiden, the Netherlands; Brigham and Women's Hospital, Harvard Medical School, United States of America

## Abstract

**Objective:**

To establish whether severe obstetric brachial plexus palsy (OBPP) can be identified reliably at or before three months of age.

**Methods:**

Severe OBPP was defined as neurotmesis or avulsion of spinal nerves C5 and C6 irrespective of additional C7-T1 lesions, assessed during surgery and confirmed by histopathological examination. We first prospectively studied a derivation group of 48 infants with OBPP with a minimal follow-up of two years. Ten dichotomous items concerning active clinical joint movement and needle electromyography of the deltoid, biceps and triceps muscles were gathered at one week, one month and three months of age. Predictors for a severe lesion were identified using a two-step forward logistic regression analysis. The results were validated in two independent cohorts of OBPP infants of 60 and 13 infants.

**Results:**

Prediction of severe OBPP at one month of age was better than at one week and at three months. The presence of elbow extension, elbow flexion and of motor unit potentials in the biceps muscle correctly predicted whether lesions were mild or severe in 93.6% of infants in the derivation group (sensitivity 1.0, specificity 0.88), in 88.3% in the first validation group (sensitivity 0.97, specificity 0.76) and in 84.6% in the second group (sensitivity of 1.0, specificity 0.66).

**Interpretation:**

Infants with OBPP with severe lesions can be identified at one month of age by testing elbow extension, elbow flexion and recording motor unit potentials (MUPs) in the biceps muscle. The decision rule implies that children without active elbow extension at one month should be referred to a specialized center, while children with active elbow extension as well as active flexion should not. When there is active elbow extension, but no active elbow flexion an EMG is needed; absence of MUPs in the biceps muscle is an indication for referral.

## Introduction

Obstetric brachial plexus palsy (OBPP) almost always involves traction of the C5 and C6 nerve roots, resulting in weakness of shoulder function and elbow flexion. Additional involvement of C7, C8 and T1 roots affects elbow extension and wrist and hand function [1], [2], [3]. The incidence of OBPP lies between 0.42–2.9 per 1000 live births [4], [5], [6]. Life-long functional impairment occurs in 20–30% of cases [7]. Mild lesions cannot be distinguished reliably from severe lesions in the perinatal period; only time reveals whether or not spontaneous recovery will occur. Early identification of severe cases facilitates early referral to specialized centers, where the need for reconstructive nerve surgery can be assessed. Identifying cases that require specialized care is challenging as no test is currently available to identify these children in the first weeks of life. Therefore, mild cases may be referred unnecessarily while severe cases may be referred too late for nerve surgery that is more effective when performed early [8]. At present, severity (based primarily on biceps function [9]) is usually assessed at 3 months of age. Lack of biceps function has been reported as an indication for nerve surgery [10], [11]. However, biceps paralysis at 3 months does not preclude a satisfactory spontaneous recovery [12], [13], [14], and establishing biceps function reliably in infants is difficult [15]. Alternative approaches to assess severity are either complex or performed at a later age [16], [17], [18]. Consequently, caretakers are often presented with overly optimistic assessments or no prediction at all, leading to parental distress [19] and treatment delays.

We aimed to develop assessment guidelines to help primary and secondary care physicians identify severe OBPP as early as possible.

## Methods

This study comprised two stages. Stage 1 was the derivation stage carried out in the Netherlands and Stage 2 was the validation stage carried out in the Netherlands and the USA. The medical ethics committees of the Leiden University Medical Center, Leiden, the Netherlands and University of Michigan Hospitals, Ann Arbor, United States of America approved the study.

### Derivation

Patients were prospectively recruited between 2002 and 2004 in the Netherlands. Infants were seen at approximately 1 week, 1 month and 3 months of age, and follow-up occurred every six months thereafter. Infants referred at 2 months or older were excluded. Ten dichotomous items concerning joint movement and needle electromyography were assessed at 1 week, 1 month and 3 months (see below). Follow-up examinations comprised testing of upper limb muscle strength, joint range of motion and function [20].

### Joint movements

Four active joint movements were examined in the supine position.

#### External shoulder rotation

The upper arm was held in internal rotation and adduction, with the elbow at 90° flexion; the hand lay on the child's abdomen. External rotation was present when the forearm was lifted from the abdomen without active elbow extension.

#### Elbow flexion

With the arm extended, flexion was present when the forearm and hand were lifted while the upper arm remained static. We did not specify a) whether flexion resulted from action of the biceps brachii muscle or the wrist extensors, b) the angle of abduction of the upper arm during flexion and c) the degree of pronation or supination. Flexion was absent when infants swung the extended arm upwards to flex the elbow.

#### Supination

With the elbow passively or actively held in 90° flexion, active rotation of the distal forearm was considered supination, regardless of flexion or extension of the wrist. When forearm rotation was effected by wrist extension and gravity, supination was considered absent.

#### Active elbow extension

With the upper arm in 90° anteflexion, active elbow extension was present if the flexed forearm could be extended regardless of the end point of the range of motion.

Shoulder abduction was not considered as a potential parameter because it remains unclear how this movement is effected in infants [21].

### Needle EMG

Needle EMG was performed on the deltoid, biceps and triceps muscles; details will be described separately. The presence or absence of spontaneous muscle fiber activity during rest and of motor unit potentials (MUPs) was scored as present or absent for each of the three muscles.

### Definition of severity

A severe lesion was defined as neurotmesis or avulsion of spinal nerves C5 and C6, irrespective of any C7-T1 lesion, assessed during nerve surgery (described elsewhere in detail [20]). Surgery was performed at four to five months of age when external shoulder rotation and active elbow flexion with supination were absent. If the presence of paralysis was indeterminate, explorative surgery was performed before six months of age to determine the severity of the lesion. A mild lesion was defined as the presence of active elbow flexion and supination at six months of age spontaneously or upon direct nerve stimulation. Patients with mild lesions showed a subtotal range of active elbow flexion, supination and abduction at two years of age.

### Validation

Two groups were prospectively studied. One group was seen in Leiden between 2005 and 2009, and the other at the University of Michigan (Ann Arbor) between 2007 and 2009. Patients were included when neurological and EMG examination could be performed at one month.

### Statistical analysis

#### Derivation

For each of the ten dichotomous items, sensitivity, specificity, positive predictive value (PPV) and negative predictive value (NPV) for the distinction between ‘mild’ and ‘severe’ cases were calculated. The optimal predictors per visit were identified with a two-step forward selection logistic regression analysis using likelihood-ratio tests with p<0.05 as the inclusion criterion. The first step comprised the four items of joint movements, and the second added the six items of the needle EMG, mimicking the clinical decision process. This analysis yielded a set of significant predictive items for each visit. For a severe lesion the estimated probability was >0.5; otherwise, it was classified as ‘mild’.

Estimated and true outcomes were used to form a 2×2 table, and the sensitivity, specificity, PPV and NPV were calculated. The proportion of correctly predicted outcomes was calculated, consisting of the sum of correctly predicted severe and correctly predicted mild lesions. This proportion was compared between the three visits. The set of predictors from the logistic regression model that resulted in the highest rate of correctly predicted results was used to develop a clinical decision rule, applied to all visits. The additional value of ancillary EMG testing for prediction after clinical testing was calculated. SPSS (version 16.0, SPSS Inc, Chicago, USA) was used.

#### Validation

In the two validation groups, the newly developed assessment guideline was used to predict mild versus severe lesions. PPV, NPV, sensitivity, specificity and the proportion of correct prediction of outcomes were calculated in both groups.

## Results

### Derivation

**Figure 1:**
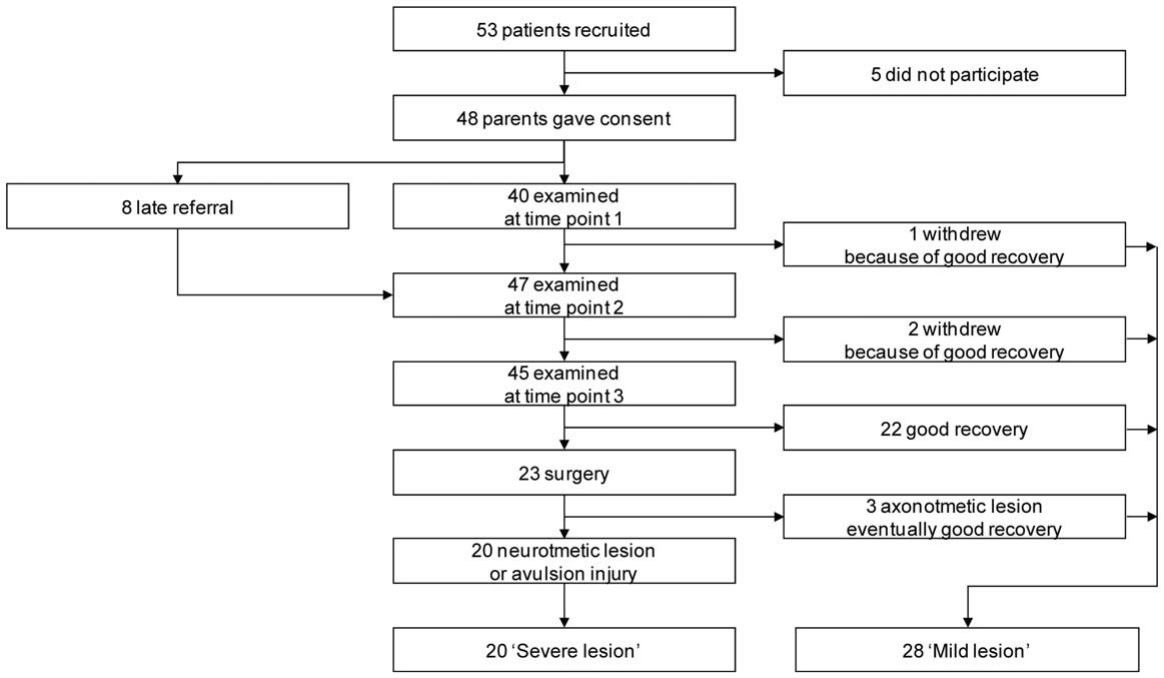
Flow diagram of tested patients. Over an eighteen month period 53 cases were contacted. The parents of five children chose not to participate. For the remaining 48 cases written informed consent was obtained. Thirty-seven of the 48 infants were seen three times. Of the remaining eleven, two were seen twice, at one week and one month, and the third visit was canceled by the parents because of good recovery. Eight were seen relatively late, so they were only seen at one and three months. One infant was only seen at one week because of good recovery afterwards. Not attended visits were regarded as missing data. The mean age at visit one was 9 days (median 9, range 12), at visit two 32 days (median 31, range 29) and at visit three 87 days (median 87, range 29).

Over an eighteen month period, caretakers of 53 patients were contacted and 48 gave written informed consent. (Figure 1) The mean age at visit one was 9 days (median 9, range 12), at visit two 32 days (median 31, range 29) and at visit three 87 days (median 87, range 29). Surgical exploration was performed in twenty-three infants. The mean age at surgery was 143 days (median 139, standard deviation (SD) 30 days). In 20 of 23 surgically treated infants, neurotmesis or avulsion of C5 and C6 was found (severe lesion, 42%). Six of the 20 infants had a pure C5, C6 lesion, seven infants had C5, C6, C7 (C8) lesions, and seven had a complete C5-T1 lesion. The three remaining operated infants and the twenty-five non-operated infants had an axonotmetic lesion. The mean follow-up was 735 days (median 704 days, SD 151 days).

### Prediction of response

The predictive value of all ten items is shown in Table 1. The highest prediction rates of the four clinical items at the three visits were as follows. Active elbow extension at visit 1 had a sensitivity of 0.70, specificity of 0.96, PPV of 0.92, NPV of 0.81. At the second visit, sensitivity was 0.55, specificity 1.0, PPV 1.0, and NPV 0.75. Elbow flexion at visit 3 had a sensitivity of 0.89, specificity 0.88, PPV 0.85, NPV 0.92.

**Table 1:** The results of the ten test items at visits one (∼1 week), two (∼1 month) and three (∼3 months).

	Visit 1				Visit 2				Visit 3			
Test result	Severe lesion(n = 17)Sensitivity	Mild lesion(n = 23)1-Specificity	p	% Corrrectly predicted (n = 40)	Severe lesion (n = 20)Sensitivity	Mild lesion(n = 27)1-Specificity	p	% Corrrectly predicted (n = 47)	Severe lesion(n = 19)Sensitivity	Mild lesion(n = 26)1-Specificity	p	% Corrrectly predicted (n = 45)
**Absence of movement**												
External rotation	17 (100%)	20 (87.0%)	0.122	20 (50%)	20 (100%)	14 (51.9%)	0.000	23 (48.9%)	19 (100%)	13 (50.0%)	0.000	32 (71.1%)
Elbow flexion	17 (100%)	13 (56.5%)	0.002	27 (67.5%)	20 (100%)	9 (33.3%)	0.000	38 (80.8%)	17 (89.5%)	3 (11.5%)	0.000	40 (88.8%)
Supination	17 (100%)	17 (73.9%)	0.022	23 (57.5%)	20 (100%)	13 (48.1%)	0.000	34 (72.3%)	18 (94.7%)	6 (23.1%)	0.000	38 (84.4%)
Elbow extension	12 (70.6%)	1 (4.3%)	0.000	34 (85%)	11 (55.0%)	0 (0%)	0.000	38 (80.8%)	7 (36.8%)	0 (0%)	0.001	33 (73.3%)
**Presence of spontaneous EMG activity**												
Deltoid	13 (76.5%)	9 (39.1%)	0.019	13 (32.5%)	17 (85.0%)	14 (51.9%)	0.018	17 (36.1%)	5 (26.3%)	1/25 (4.0%)	0.033	15/44 (34.0%)
Biceps	9 (52.9%)	8 (34.8%)	0.251	16 (40%)	18 (90.0%)	8 (29.6%)	0.000	10 (21.2%)	7 (36.8%)	3/25 (12.0%)	0.051	15/44 (34.0%)
Triceps	11 (64.7%)	4 (17.4%)	0.002	10 (25%)	15 (75.0%)	5 (18.5%)	0.000	10 (21.2%)	4 (21.1%)	2/25 (8.0%)	0.211	17/44 (38.6%)
**Absence of MUPs**												
Deltoid	15 (88.2%)	9 (39.1%)	0.002	29 (72.5%)	20 (100%)	9 (33.3%)	0.000	38 (80.8%)	5 (26.3%)	1/25 (4.0%)	0.033	29/44 (65.9%)
Biceps	15 (88.2%)	7 (30.4%)	0.000	31 (77.5%)	20 (100%)	5 (18.5%)	0.000	42 (89.3%)	1 (5.3%)	0/25 (0%)	0.246	26/44 (59.0%)
Triceps	10 (58.8%)	1 (4.3%)	0.000	32 (80%)	10 (50.0%)	0 (0%)	0.000	37 (78.7%)	1 (5.3%)	1/25 (4.0%)	0.842	25/44 (56.8%)

For each of the ten dichotomous items concerning joint movements and needle electromyography, the sensitivity, 1- specificity and percentage of correct prediction of a ‘mild’ or ‘severe’ lesion is indicated. Electromyography (EMG): the presence of spontaneous muscle activity (fibrillation and/or positive sharp waves) and the absence of motor unit action potentials (MUPs). p values denote results from Pearson's Chi-Square test.

Logistic regression analysis at 1 week of age identified only 1 significant parameter for severity in the first selection step: the presence or absence of active elbow extension. The second step added the presence or absence of MUPs in the deltoid muscle. This model correctly predicted the outcome in 85% (34/40) of cases (sensitivity 0.70, specificity 0.96).

**Figure 2:**
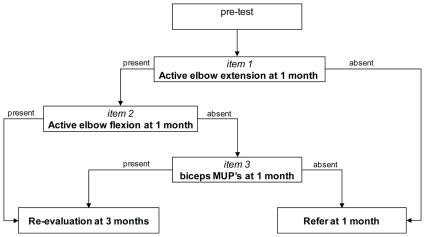
Flow diagram of OBPP assessment at one month of age using the Leiden three item test. Prediction at one month of age was better than at one week and three months. The decision rule implies that children without active elbow extension at one month should be referred, while children with active elbow extension as well as flexion should not. When there is elbow extension, but no active elbow flexion an EMG is needed; absence of MUPs in the biceps muscle is a reason for referral.

At one month of age, three items were selected: elbow extension, elbow flexion and MUPs in the biceps (Figure 2). These three items individually had correct prediction rates of 80.8%, 80.8% and 89.3%. The logistic model using these items predicted the outcome correctly in 93.6% (44/47) of infants (sensitivity 1.0, specificity 0.88, PPV 0.87, NPV 1.0). Clinical testing of extension and flexion at one month, without performing an EMG of the biceps muscle, resulted in a prediction rate of 80.8%. (sensitivity 1.0, specificity 0.66, PPV 0.68, NPV 1.0). EMG increased the percentage of correct predictions by 13%.

At three months of age, the selected variables were elbow flexion and supination. This model correctly predicted outcome in 88.8% (40/45) of infants (sensitivity 0.94, specificity 0.88).

Since the model of the second visit had the highest prediction rate, we used this model to derive a simple assessment guideline: the Leiden three item test (Figure 2).

### Validation

**Figure 3:**
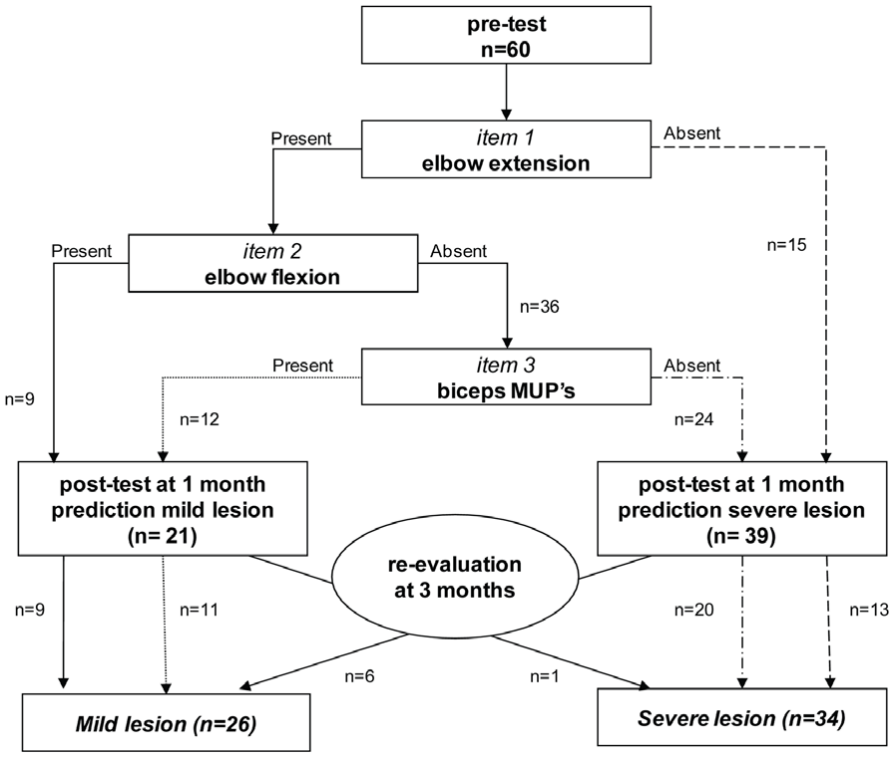
Flow diagram of LUMC validation group (n = 60). Follow-up data resulted in a severe lesion in 34 infants (57%). The three item test indicated a severe lesion in 39 infants (65%). The test predicted outcome correctly in 88% (53/60) of infants (sensitivity 0.97, specificity 0.76, PPV 0.84, NPV 0.95. The dash style of the arrows indicates related patient flows.

325 OBPP infants were routinely referred to the LUMC; the vast majority was referred later than one month and was excluded. Sixty patients were included with a mean age at testing of 31 days (median 30, range 18). Follow-up showed severe lesions in 34 infants (57%). The three item test indicated a severe lesion in 39 infants (65%), with a sensitivity of 0.97, specificity 0.76, PPV 0.84, NPV 0.95 (Figure 3). The proportion of correctly predicted outcomes was 88.3% (53/60). Limiting the test to extension and flexion examination at one month resulted in a correct prediction rate of 71.6% (sensitivity 1.0, specificity 0.34, PPV 0.66, NPV 1.0). EMG testing increased correct prediction by 17%.

Forty-five OBPP infants were referred to the University of Michigan, of which 13 met the inclusion criteria. Mean age at testing was 31 days (median 33, range 18). A severe lesion was found in 7 (54%). The three item test indicated severe lesions in 9 (69%). The test predicted outcome correctly in 84.6% (11/13) with a sensitivity of 1.0, specificity 0.66, PPV 0.77, NPV 1.0. The combination of extension and flexion testing at one month resulted in a correct prediction in 76.9% (sensitivity 1.0, specificity 0.50, PPV 0.70, NPV 1.0). EMG testing increased correct prediction by 8%.

## Discussion

We aimed to identify robust parameters to assess the severity of OBPP in a large prospective series of infants at an early age. An assessment strategy was developed and validated in two cohorts of infants. The best predictor of a severe lesion was achieved at one month of age, based on three items: active elbow extension, active elbow flexion and needle EMG of the biceps muscle. The rate of correct predictions was excellent in the derivation group at 94%, with a sensitivity of 1.0 and specificity of 0.88. In both validation groups, the correct prediction rate was slightly lower at 88% and 84%. Sensitivity was similarly high, but specificity was slightly lower.

### Clinical consequences

We advise that infants with OBPP, who fulfill the criteria for a severe lesion according to the Leiden three-item test at one month of age, should be referred to a specialized center (see figure 2). This strategy has advantages: (1) minimization of delays that contribute to the deleterious effects of prolonged denervation; (2) caretakers can be informed promptly regarding prognosis and treatment; (3) the first 2 items of the three item test guides primary-care physicians when to request needle EMG of the biceps muscle.

The three item test was slightly pessimistic, as a small number of patients with an abnormal test showed late spontaneous recovery. We would contend that this error is more desirable than the opposite one in which infants with severe lesions are recognized too late. Monitoring of the progress and speed of recovery in the 2nd and 3rd months is strongly advised. When spontaneous recovery does not occur in this time frame, the detailed information of the three item test acquired at one month provides adequate rationale to perform CT-myelography to detect root avulsions [22].

Active elbow extension emerged as a significant predictor of a C5 and C6 lesion. Although the triceps muscle is largely innervated through C7 and C8 roots, a possible reason for this apparent oddity is that the C5 and C6 roots are virtually always affected in OBPP, while the more caudal C7, C8 and T1 roots are only affected in more extensive lesions [3]. Paralysis of elbow extension likely acts as a proxy for severe lesions of C5 and C6 roots.

### Application of the test

In routine practice, examiners generally test both active elbow flexion and extension. When active elbow flexion and extension are clearly present, no EMG is necessary, but reticence to perform an EMG should not be a barrier. In our practice, the procedure of EMG, if explained properly, is borne well by infants as well as parents.

Unexpectedly, the predictive value of the three-item test was better at one month than at three months of age. The slow development of spontaneous functional recovery suggests that recovery becomes clearer the later a child is examined. The superiority of prediction at one month rests on the contribution of the EMG at one month, but not at three months. An apparent paralysis of the biceps at three months of age is almost always accompanied by the paradoxical presence of MUPs in that muscle [23]. In OBPP, the C5 and C6 spinal nerves are rarely completely ruptured. Instead a “neuroma in continuity” is present. A small percentage of severed axons may advance past this neuroma, reflected by the appearance of MUPs at three months. Reasons for the lack of a clinical counterpart have been discussed [23]. The presence of MUPs at one month of age likely suggests that these axons were previously dysfunctional due to neurapraxia but not axonotmesis, thereby carrying a better prognosis.

### Limitations

We actively recruited cases for the derivation study that probably affected the proportions of mild and severe cases, but this does not affect the validity of the Leiden three item test. Assessment of severity was not rigidly blinded, but severity was assessed at around 150 days of age when earlier data were not reviewed. Combined with the applied way of assessment, we do not feel that this factor significantly influenced the results.

Selection of severe cases involved selection of those for nerve surgery and assessment of surgical findings. Follow-up in the derivation group did not show any severe cases among infants who had not undergone surgery, so the two-step procedure did not introduce errors.

Finally, there is no widely accepted definition for the severity of OBPP [24]. We feel that the definitions used here do justice to the purpose of our study.

### Conclusion

The severity of OBPP can be reliably predicted at one month of age in the majority of infants with OBPP by testing active elbow extension, active elbow flexion and recording MUPs in the biceps muscle. The Leiden three item test can be implemented in routine clinical practice to identify those infants with OBPP who require prompt referral to specialized centers.
